# Frequent respiratory viral infections in a young child in a 27-month follow-up study

**DOI:** 10.1099/jmmcr.0.003020

**Published:** 2014-12-01

**Authors:** Atsushi Kaida, Hideyuki Kubo, Nobuhiro Iritani, Seiji P Yamamoto, Atsushi Hase, Koh-Ichi Takakura, Tsutomu Kageyema

**Affiliations:** ^1^​ Department of Microbiology, Osaka City Institute of Public Health and Environmental Sciences, 8-34 Tojo-cho, Tennoji-ku, Osaka 543-0026, Japan; ^2^​ Department of Biological Resources Management, School of Environmental Science, The University of Shiga Prefecture, 2500 Hassaka-cho, Hikone-City, Shiga 522-8533, Japan; ^3^​ Influenza Virus Research Center, National Institute of Infectious Diseases, Gakuen 4-7-1, Musashimurayama-shi, Tokyo 208-0011, Japan

**Keywords:** detection duration, frequency, nursery school, respiratory virus infection, young children

## Abstract

**Introduction:**

Viruses are major aetiological agents of acute respiratory infection in young children. Although many studies have reported detection and analysis of respiratory viruses in sporadic cases, there have been few follow-up studies of individuals. The purpose of this study was to investigate the frequency of respiratory viral infections in a young child and to examine the duration of viral genome detection in clinical specimens.

**Case presentation:**

A total of 284 nasal swabs were collected during symptomatic (196 specimens) and asymptomatic (88 specimens) periods of respiratory symptoms from a young female child (from 4 months to 31 months of age, who was admitted to a nursery school at 9 months). Multiplex real-time PCR for 19 respiratory viruses or subtypes was performed. One hundred and ninety-eight of the tested specimens were virus positive (69.7 %) (symptomatic periods, 149/196, 76.0 %; asymptomatic periods, 49/88, 55.7 %). Rhinovirus was the most frequently detected (26 times). Long durations of detection were observed for human coronavirus NL63 (30 days), rhinovirus (28 days) and human bocavirus 1 (22 days).

**Conclusion:**

Young children living in a group context have a high risk of respiratory virus infections, especially rhinovirus. In some instances, viral genomes were detectable for about 1 month by PCR.

## Introduction

Over 200 serologically or genetically different viruses have been identified as major aetiological agents of acute respiratory infections (Heikkinen & Jarvinen, 2003; Eccles, 2005). Although many viral infections are limited to the upper respiratory tract, viral infections of the lower respiratory tract can cause serious symptoms in children (Henrickson *et al.*, 2004; Pavia, 2011).

Many studies have reported and identified respiratory viruses in sporadic cases of acute respiratory infection, but there have been few follow-up studies of individuals with multiple respiratory virus infections (Martin *et al.*, 2013). Thus, it is unclear how many types of respiratory viruses can infect a single person. We conducted a 27-month study to investigate the frequency of infection of 19 different respiratory viruses and subtypes in a young child in a nursery school. We sought to determine the duration of viral genome detection in clinical specimens using multiplex real-time PCR.

## Case report

Between November 2010 and January 2013, 284 nasal swabs were collected from an immunocompetent female child (no underlying disease with no tobacco smoke exposure; living with father, mother and one brother (+3 years of age compared with the index child) in the same household in a city. The child was 4 months of age at the start of the study and was admitted to a nursery school at 9 months of age. The brother was also admitted to the same nursery school during the study period. About 15 children were present in the same room, and about 140 children (0–6 years old) were present in the nursery school.

One hundred and ninety-six swabs were taken during symptomatic periods when the child showed some clinical signs such as rhinorrhoea, phlegm, cough and/or fever. The remaining 88 swabs were collected during asymptomatic periods. Informed consent was given by the parents for collection of specimens and analysis of viruses present in the specimens. Specimens were collected by the parents in the home and stored at −20 °C before testing. During the symptomatic period, at least two specimens were collected each week. When no respiratory symptoms were observed, a specimen was collected every 1 or 2 weeks. When specimens collected within 7 days of each other were consecutively virus positive, the child was judged to be virus positive for the entire period between sampling.

Viral RNA was extracted using a QIAamp Viral RNA Mini kit (Qiagen), and cDNA synthesis was performed using SuperScript III (Life Technologies) as described previously (Kaida *et al.*, 2011). Multiplex real-time PCR for detection of respiratory viruses and subtypes comprising human metapneumovirus (hMPV), respiratory syncytial virus (RSV) (A and B), human parainfluenza virus types 1–4 (HPIV-1–4), human bocavirus 1 (HBoV1), human coronavirus (HCoV) (229E, OC43, HKU1 and NL63), influenza virus [A (FLUAV), A (H1N1) 2009, B (FLUBV) and C (FLUCV)], human adenovirus (HAdV), human enterovirus (HEV) and human rhinovirus (HRV) (A, B and C) was performed using a QuantiTect multiplex PCR kit (Qiagen). Primers and TaqMan probes and PCR conditions were as described by Kaida *et al.* (2014).

A total of 198 (69.7 %) specimens were found to be virus positive. The number of virus-positive samples was higher during symptomatic periods (149/196; 76.0 %) compared with during asymptomatic periods (49/88; 55.7 %) (Fisher’s exact test, *P*<0.001). Of the 198 virus-positive specimens, 132 (66.7 %) were positive for a single virus, whereas the remaining 66 (33.3 %) were positive for more than one virus type. A slightly higher ratio of multiple/total virus-positive specimens was collected during the symptomatic period (52/149; 34.9 %) than the asymptomatic period (14/49; 28.6 %) (Fisher’s exact test, *P* = 0.486).

A total of 13 viruses and subtypes were detected during the study period. The total number of detections (co-detections) of each virus were: HRV, 131(58); HAdV, 36 (24); HBoV1, 32 (24); HEV, 14 (four); HCoV-NL63, 13 (nine); HCoV-OC43, eight (two); hMPV, eight (four); FLUCV, seven (five); HPIV-3, seven (two); HCoV-HKU1, six (two); RSV A, four (one); FLUAV, three (two); and HPIV-4, three (three). The combinations of viruses co-detected in 66 samples are shown in Table 1. Only HRV was detected before the child was admitted to nursery school (3/24, 12.5 %). The HRV positivity rate increased after admission to nursery school (128/260, 49.2 %) when compared with the positivity rate prior to admission (Fisher’s exact test, *P*<0.001).

**Fig. 1.**
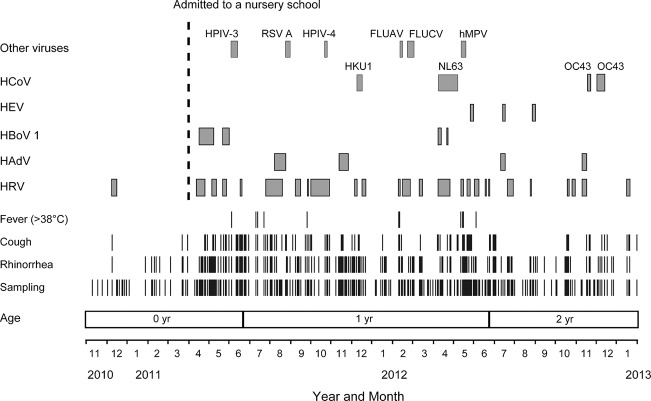
Results of the 27-month follow-up study of respiratory virus infections and the duration of detection. A schematic distribution of the respiratory viruses and the duration of detection are shown.

The distribution and duration of virus detection and clinical signs are shown in Fig. 1. Rhinorrhoea, cough and fever were recorded at 66.7, 38.8 and 7.3 % of the sampling dates, respectively. The most frequently detected virus was HRV (26 occasions), followed by HAdV (four), HBoV1 (four), HEV (three), HCoV-OC43 (two), HCoV-HKU1 (one), HCoV-NL63 (one), hMPV (one), HPIV-3 (one), HPIV-4 (one), FLUAV (one), FLUCV (one) and RSV A (one). The longest duration of virus detection was observed for HCoV-NL63 (30 days). The duration of detection for the other viruses was as follows: HRV (3–28 days), HBoV1 (2–22 days), HAdV (7–17 days), HEV (4–5 days), HCoV-OC43 (5–12 days), FLUCV (11 days), HPIV-3 (11 days), HCoV-HKU1 (9 days), hMPV (8 days), RSV A (8 days), FLUAV (6 days) and HPIV-4 (5 days).

## Discussion

Although some follow-up studies of respiratory viral infections have been reported, there are no reports describing the detection and analysis of 19 different respiratory viruses from numerous specimens collected from one person (Martin *et al.*, 2013; Kawabuchi–Kurata *et al.*, 2014).

The virus positivity rate was higher in specimens collected after the child was admitted to nursery school (195/260, 75.0 %) than before she was admitted (3/24, 12.5 %) (Fisher’s exact test, *P*<0.001). These results indicate a high risk of respiratory virus infection in young children in a group context. It has been reported that young children (<1.5 years of age) in daycare have a higher risk of upper respiratory virus infections compared with homecare children (Lu *et al.*, 2004).

HRV was detected most frequently throughout the study period, suggesting that there is a high risk of infection with this virus in young children. HRV is already known to be one of the most frequently detected respiratory viruses in children (Monto & Sullivan, 1993; Martin, Fairchok *et al.*, 2013). There are over 100 serotypes of HRV, which would contribute to the frequent repeated infections in young children (Bochkov *et al.*, 2011). Recent studies have also shown that HRV is associated with lower respiratory tract infections, and it is one of the most important respiratory tract pathogens (Mackay, 2008). HRV infection may not be highly associated with fever as it was only present at a low rate on HRV-single-positive occasions (11.5 %, 3/26). In fact, in a previous study concerning febrile respiratory tract infections in Japanese paediatric outpatients, HRV was detected in 4.6 % of tested specimens, excluding influenza virus-positive patients (Hara *et al.*, 2014). Despite being one of the most frequent respiratory virus infections for young children, most cases only experience mild symptoms.

In addition to HRV, HAdV, HBoV1, HEV and HCoV-OC43 were also detected multiple times. Circulating viruses may infect the same person repeatedly when young children live in group contexts. HBoV1 was detected four times during the study period, and the duration of detection decreased with repeated infection, which may have been related to the child’s immunity to HBoV1. The data also suggest that HBoV1 might exist in a latent state after infection and be reactivated by other respiratory viral infections (Jula *et al.*, 2013). A precise genomic analysis of the HBoV1 strains isolated in this study would enhance our understanding of the reasons underlying repeated detection of HBoV1.

Because HAdV and HEV consist of multiple serotypes, frequent infection may be due to different serotypes. The reason for repeated detection of HCoV-OC43 is uncertain.

Some viral genomes were detected for approximately 1 month in this study. This result supports previous reports of long-term viral shedding of HRV, HEV and HBoV1 (Martin *et al.*, 2010, 2013; Li *et al.*, 2013). To our knowledge, this is the first report of the long-term (30 days) detection of HCoV-NL63. Young children have not yet obtained adaptive immunity to many viruses and their immature immune systems may lead to a delay in the elimination of pathogens from the body, thereby resulting in long-term viral genome detection.

Respiratory viral co-infections in children have been well documented (Stempel *et al.*, 2009; Zhang *et al.*, 2012). In addition, many respiratory viruses have been detected in asymptomatic children (Jartti *et al.*, 2008; Advani *et al.*, 2012). A follow-up study for a young child in this study also showed a high proportion of multiple virus-positive specimens in both symptomatic and asymptomatic periods, supporting previous findings. However, the effect of multiple viral infections on the severity of clinical signs is uncertain as described previously (Goka *et al.*, 2013).

This study was very limited because only one child was sampled and homecare children were not examined for comparison. Virus detection by PCR was not always associated with clinical symptoms, and the effect of bacterial infections is uncertain. Despite the limitations of our study, this long-term follow-up research demonstrated that one young child was infected by many respiratory viruses when living in a group context. This study also provides baseline data that demonstrate that young children have a high risk of respiratory virus infection, particularly HRV, in nursery schools. A follow-up study of more children, with precise viral identification (typing), analyses of clinical symptoms and a consideration of bacterial detection, would be helpful to understand respiratory infections in young children. Additional epidemiological data may also help to prevent severe respiratory virus infections and facilitate the development of a vaccine(s).

**Table:** Table 1. Number of co-detections for each respiratory virus

Virus	HAdV	HBoV 1	HCoV-NL63	HCoV-229E	HCoV-OC43	HCoV-HKU1	HEV	HPIV-1	HPIV-2	HPIV-3	HPIV-4	hMPV	HRV	FLUAV*	FLUA (H1N1) 2009	FLUBV	FLUCV	RSV A	RSV B
HAdV		–	–	–	–	–	2	–	–	–	–	–	17	–	–	–	2	1	–
HBoV 1	–		1	–	–	–	1	–	–	1	–	–	15	–	–	–	–	–	–
HCoV-NL63	–	6		–	–	–	–	–	–	–	–	–	2	–	–	–	–	–	–
HCoV-229E	–	–	–		–	–	–	–	–	–	–	–	–	–	–	–	–	–	–
HCoV-OC43	–	–	–	–		–	–	–	–	–	–	–	2	–	–	–	–	–	–
HCoV-HKU1	–	–	–	–	–		–	–	–	–	–	–	2	–	–	–	–	–	–
HEV	–	–	–	–	–	–		–	–	–	–	–	1	–	–	–	–	–	–
HPIV-1	–	–	–	–	–	–	–		–	–	–	–	–	–	–	–	–	–	–
HPIV-2	–	–	–	–	–	–	–	–		–	–	–	–	–	–	–	–	–	–
HPIV-3	–	–	–	–	–	–	–	–	–		–	–	1	–	–	–	–	–	–
HPIV-4	–	–	–	–	–	–	–	–	–	–		–	3	–	–	–	–	–	–
hMPV	2	–	–	–	–	–	–	–	–	–	–		2	–	–	–	–	–	–
HRV	2	6	6	–	–	–	–	–	–	–	–	2		2	–	–	3	–	–
FLUAV*	–	–	–	–	–	–	–	–	–	–	–	–	–		–	–	–	–	–
FLUA (H1N1) 2009	–	–	–	–	–	–	–	–	–	–	–	–	–	–		–	–	–	–
FLUBV	–	–	–	–	–	–	–	–	–	–	–	–	–	–	–		–	–	–
FLUCV	–	–	–	–	–	–	–	–	–	–	–	–	–	–	–	–		–	–
RSV A	–	–	–	–	–	–	–	–	–	–	–	–	–	–	–	–	–		–
RSV B	–	–	–	–	–	–	–	–	–	–	–	–	–	–	–	–	–	–	

Upper right shows the number of dual co-detections with each respiratory virus; lower left shows the number of triple or more co-detections with each respiratory virus.

–, Not detected.

* FLUAV: Values were counted as number of FLUAV-positive detections minus number of FLUA (H1N1) 2009-positive detections.

## Abbreviations

HAdV: human adenovirus
HBoV: human bocavirus
HCoV: human coronavirus
HEV: human enterovirus
HPIV: human parainfluenza virus
hMPV: human metapneumovirus
HRV: human rhinovirus
FLUAV: influenza virus A
FLUBV: influenza virus B
FLUCV: influenza virus C
RSV: respiratory syncytial virus
